# Smart Contract Centric Inference Engine For Intelligent Electric Vehicle Transportation System

**DOI:** 10.3390/s20154252

**Published:** 2020-07-30

**Authors:** Prince Waqas Khan, Yung-Cheol Byun

**Affiliations:** Department of Computer Engineering, Jeju National University, Jeju-si 63243, Korea; princewaqas12@hotmail.com

**Keywords:** electric vehicles, transportation systems, blockchain, sensors, actuators, vehicle control unit, smart contract, machine learning, LSTM, stator temperature

## Abstract

The provision of electric vehicles (EVs) is increasing due to the need for ecological green energy. The increment in EVs leads to an intelligent electric vehicle transportation system’s need instead of cloud-based systems to manage privacy and security issues. Collecting and delivering the data to current transportation systems means disclosing personal information about vehicles and drivers. We have proposed a secure and intelligent electric vehicle transportation system based on blockchain and machine learning. The proposed method utilizes the state of the art smart contract module of blockchain to build an inference engine. This system takes the sensors’ data from the vehicle control unit of EV, stores it in the blockchain, makes decisions using an inference engine, and executes those decisions using actuators and user interface. We have utilized a double-layer optimized long short term memory (LSTM) algorithm to predict EV’s stator temperature. We have also performed an informal analysis to demonstrate the proposed system’s robustness and reliability. This system will resolve the security issues for both information and energy interactions in EVs.

## 1. Introduction

The transportation sector is considered one of the largest sources of greenhouse gas emissions and causes climate change and harms the air quality index. One possible solution to this problem is switching to electric vehicles (EVs) instead of fossil fuel-based vehicles [1]. Conventional fuel vehicles have an internal combustion engine. Electric vehicles have electric motors. Therefore, it is considered environmentally friendly because it does not emit exhaust gases. There are different electric motors such as direct current (DC) series motor, brushless DC motor, and permanent magnet synchronous motor (PMSM) [2]. Some operators and public charging stations are offering free charging to attract more and more customers. In this case, it does not require implementing a payment solution. Many companies have developed solutions based on their systems, which rely on closed digital membership and operator cards for operators and manufacturers of prepaid cars. These methods are minimal and cannot meet the broad market demand. In order to ensure a comfortable experience for all EV owners, payment methods must meet specific basic requirements, such as safety, comfort, and globalization. In some countries, payment systems have strong anti-fraud laws, but laws are not reliable in some states. Some countries do not accept the laws of other countries. In this regard, modern information technology can help adapt to the benefit of electricity to these innovative changes and meet consumer demand. The increment in global positioning system enabled vehicles and, based on real-time location data acquired from them, has led to the need for prevalent location-dependent solutions [3]. Collecting and providing data to an intelligent transportation system (ITS) means also exposing the privacy of vehicles and their drivers. Currently, its architectures and services are based on centralized clouds where vehicles and users are identified, authorized, and authenticated, and services are provided [4]. The malicious behaviors from both service providers or other users cannot be well traced and found in time [5].

Blockchain technology is developing rapidly, and practical applications have begun in many fields such as logistics [6], medical [7], and food safety [8]. It is foreseeable that more and more industries will apply them in the future. Blockchain meets consumer trust and privacy issues, so it is reliable and highly recommended for use in modern society. The main feature of blockchain is smart contracts. A smart contract is a digital contract form between two or more parties [9]. We have proposed a smart contract centric inference engine for an intelligent electric vehicle transportation system (IEVTS). This system utilizes the sensor data acquired from the vehicle control unit (VCU) and makes decisions using the inference engine. In this system, users can manage their personal and EV information. We have used the Hyperledger Fabric, an open-source platform by IBM. This is an easy-to-use platform for blockchain systems [10]. The Hyperledger Fabric’s smart contract feature allows developers to define a set of applicable rules automatically. When the smart contract is executed, it automatically processes the encrypted rules. The primary contributions of this article are:

- We provide a smart EV management system, an intelligent electric vehicle transportation system (IEVTS) equipped with a smart contract. The smart contract allows customers to register and sell transactions related to electric vehicles without disclosing personal information.
- We use blockchain to provide transparency and reliability of the EV management system.
- Machine learning-based algorithm, optimized LSTM has been applied to the prediction of stator temperature.

The rest of this article first discusses the latest developments in electric vehicles and blockchain. Then we give an overview of the design and architecture of the proposed model. We evaluate the operation of the proposed model using different results. This article ends with a few discussions and conclusions.

## 2. Related Work

Fossil fuel-based vehicles are one of the significant reasons for greenhouse gas emissions and climate changes. Many security and privacy concerns are related to electrical vehicle networks, making it challenging to use existing centralized systems. The modern-day solution for the secure database is blockchain. Blockchain is a ledger that securely and anonymously tracks all transactions. Many researchers are using blockchain in the area of vehicle transportation systems [11]. This section provides the preliminaries and related work in the domain electric vehicle using blockchain technology and smart contracts. Hyperledger Fabric provides a user-friendly production environment. It gives the user the ability to maintain log operations, enhanced monitoring, and management of nodes. It also provides an easier-to-use programming model and development interface [12]. The process of making a chain starts with the initialization of a transaction by the node in the chain. Then these transactions are merged into blocks. The consensus algorithm checks the block. After reviewing the block, the block becomes part of the tamper-resistant chain. All transactions are time-stamped, and it is hard to tamper with the transaction. The consensus must satisfy two characteristics: safety and vitality to ensure the same knot. Security means that each node provides the same input sequence and produces the same output on each node. When a node receives the same set of transactions, the same state change occurs on each node [13]. Dynamic means that all nodes without faults will eventually receive all committed transactions unless the connection fails.

Ho et al. [14] use blockchain to check information on the internet of vehicles (IOV). They use the Byzantine consensus algorithm to verify the nodes and establish consensus between the peer nodes based on time and gossip protocol. Their experimental results show that the proposed solution provides the security of communication. It also improves the effectiveness of reaching consensus among peers. Knirsch et al. [15] use blockchain technology to set up a series of charging stations to maintain confidentiality. He proposed a protocol to find an ideal charging station that could provide an overview of blockchain-based queries. Personal information such as a customer’s location is not revealed during the execution of the agreement. Chaudhary et al. [16] introduced a blockchain-based ITS for secure energy trading. In particular, this solution uses a software-defined network (SDN) as the basic structure. In this scheme, cryptocurrency is used to exchange energy in the private blockchain. Miner nodes are determined based on various factors, such as power demand, price, and downtime. It makes it difficult for attackers to be part of modification in the node selection process. Proof of work (pow) was also introduced to confirm energy transactions. According to the performance analysis, their proposed solution is lightweight and minimizes network resources’ communication and computer costs. The also proved that SDN architecture supplements the blockchain by improving the quality of service (QoS) of the network. However, they did not mention the flow control mechanisms of SDN.

Hanada et al. [17] introduce an internet of things-based application, which uses smart contracts to add gasoline to the device. They proposed a system for payment transactions using smart contracts. The developed method is based on Ethereum. As long as users have a blockchain network, this system allows users to monitor all interactions between the system and the blockchain platform. Automated gasoline purchases eliminate third parties involved in the payment system and save up to 79% in transaction fees compared to traditional methods. The chargeitup case study was proposed by Pedrosa  et al. [18] to analyze the fuel condition of autonomous vehicles. The proposed system is based on Ethereum and is mainly used to send machine-to-machine transactions using smart contracts. They presented the full architecture of a state channel for smart mobility systems. Liu et al. [19] focus on information security for electric vehicles, energy interaction in cloud computing, and edge computing. Depending on the different roles of electric vehicles, they offer attractive vehicle applications. They describe blockchain-influenced data and energy cryptocurrencies to provide evidence of distribution consistency, frequency of data sharing, and specific energy contributions. Security solutions for edge and cloud computing are proposed to provide an intuitive understanding of automotive applications.

Electrical vehicle charges should be distributed fairly and equitably across the charging stations to avoid load transfer and switch devices. In an article by Jin et al. [20] introduces blockchain-specific business methods and models. Considering the various random factors of the charging station, the Monte Carlo model was used to specify the charging station requirements. Depending on the charging station’s loading requirements, the charging station must make future deliveries. Blockchain assigns reasonable payment rights and guarantees the security of convenience. It is not connected to a charging station or any other source of energy in the existing network. The adaptive blockchain-based electric vehicle method is proposed to reduce the level of power fluctuation by Liu et al. [21]. The iceberg demand algorithm is used to implement a sample demand strategy.it helps to adapt to network loading and unloading systems. Ethereum-based platforms are used, and they can achieve lower consumption costs compared to other Ethereum-based EV systems. The author had presented the new charging scheme with a capable decentralized blockchain-enabled smart grid system. Their goal is to reduce the conversion rate of electricity into the grid and recoup the costs of electrical consumers. However, they didn’t focus on striking a balance between the complexity of the off-chain and the complexity of on-chain while still using the blockchain’s decentralized function.

In the internet of things scenario, an excellent solution for consumers, rich-thin-clients IoT solution is created by Sun et al. [22], using the limited resources of the internet of things tools while implementing blockchain mining methods. Both rich and thin clients can access blockchain and data collection operations, and only rich clients can perform mining operations. According to the solution, the battery replacement method also provides the preferred fuel system for electric vehicle batteries. Besides, experience clarifies the principle of solution. However, they used a raspberry layer to mimic the original scheme. Zhou et al. [23] provide a contract-based approach to encourage electric vehicles to respond in demand response. They merge the functions of the blockchain consortium and the theoretical model of agreement. They also use computing intelligence to establish a secure and energy-efficient trading framework. A solution is suggested to use blockchain technology to safely schedule the charging system by Javed et al. [24]. They suggested vehicles to vehicles and vehicle to grid charging strategy. They also conducted simulations to demonstrate the effectiveness of the proposed work. In work by Zheng et al. [25], the aggregated based optimization model was introduced. Given the random characteristics of the charging procedure, they use genetic algorithms to explain the parameters in the system model. However, it should be noted that the individual EV load/discharge algorithms mentioned above provide constant settings for the current EV charge time. Renewable energy sources such as solar photovoltaic can be used appropriately to meet electric vehicles’ high energy demands. Electrical devices can also be used to provide additional energy-saving environments such as energy storage, spin storage, frequency, and voltage control. The biggest challenge is installing charging stations for renewable energy, power pipes, and electrical devices connected to telecommunications. Madhu et al. [26] propose a new framework for installing electronic devices in social charging stations using solar cells and networks. Besides, electric network vehicles provide additional power for frequency control. The proposed intelligent power management console is used to take full advantage of solar energy and determine the operating condition, such as network connection condition or partial condition. However, it did not provide any probable analysis and performance results for the proposed system.

Zyskind et al. [27] suggested that blockchain can be used to create decentralized personal data management systems. The authors designed two types of non-monetary transactions, assuming that they would individually access the data on the blockchain and write the data to the ledger. Access to personal information is monitored through blockchain. When a user attempts to access the database to store or retrieve data, and access transaction is sent. After agreeing to access the transaction, the user must specify the data transaction requirements to terminate the connection between the blockchains. However, overall and performance issues were not considered. Lei et al. [28] propose a new key management proposal to address the significant changes among security managers in heterogeneous vehicle communication system. They introduced the concept of blockchain in periods of dynamic transfer to optimize the transition. The proposed structure allows the safe transfer of keys through a distributed security engine. They have developed an efficient and flexible method for determining the transfer time to reduce the time of large-scale blockchain-based shipment. However, they did not pay attention to the pseudonym management. The ability to collect various environmental and administrative data from equipment for the efficient and economical operation of current and future intelligent transportation systems is critical. However, due to the privacy and security restrictions of the existing ITS system, users and devices cannot provide information. Li et al. [5] also used blockchain technology’s new ITS architecture to address privacy and security issues and encourages users and devices to provide information to ITS. The proposed blockchain architecture is used as a trust infrastructure to maintain user privacy and provide reliable services to customers. It is also compatible with the legacy of ITS infrastructure and services. Demir et al. [29] provides a tamper-free ledger for vehicle insurance records. The proposed insurance registration system covers all aspects of insurance transfer. This not only improves the insurance experience but also serves as a guide when disputes arise. The ledger can provide comprehensive services on providing clean records. This blockchain-based solution includes all stakeholders from individual drivers, dealers, insurance companies, lawyers, law enforcement agencies, and car agents. In their work however, they have not mentioned the technological aspect of the automotive industry.

In contrast to the above methodology, this article aims to study the management model of electric vehicle management based on the blockchain ecosystem. The proposed task uses smart contract technology to build an inference engine that has not yet been addressed in the current articles.

## 3. Proposed System Model

**Figure 1:**
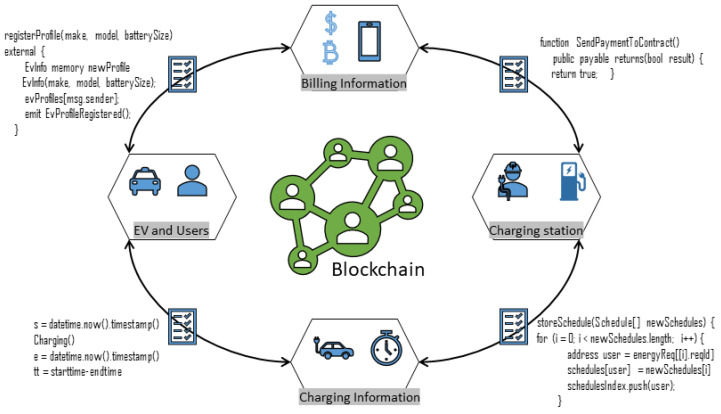
Integration scenario for different actors of blockchain based IEVTS.

The proposed system has five main contributors. The first is the administrator, the second user, or owner of the electric vehicle. The third is the charging station manager, and the fourth is the insurance agent. The latter is a regulator. All participants must create a blockchain network account using the certificate authority. The service provider assigns each user a unique private key. Electronic vehicle information is also stored in the blockchain. Figure 1 shows the integration scenarios for different actors of the blockchain-based proposed intelligent electric vehicle transportation system (IEVTS). All billing and charging information is stored in the blockchain using smart contracts. A smart contract will act as an inference engine to make decisions. It will take input from the different users and sensors, and then it will analyze the input. The input data will be used to make different decisions according to predefined rules. Then these decisions can be implemented through actuators or mobile and desktop applications. Managers and producers can check the payments of registered users. After the motor user has access to the network, the pump manager can access the EV user data. These privileges may be regulated in violation of the smart protocol access control policies to protect user-information confidentiality and integrity. Measuring the torque, rotor, and stator temperatures of the electromotor is not reliable nor economically feasible in commercial applications. Hence, we have also proposed the machine learning algorithm to predict the motor temperature.

### 3.1. System Flow

The proposed system is based on a smart contract centric inference engine, which can make decisions without human interaction. The smart contract is a set of commitments defined in digital form, and it includes agreements that contract participants can execute [30].

**Figure 2:**
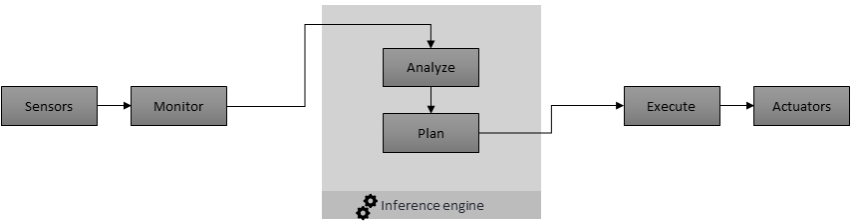
Inference engine based system flow.

A smart contract-based inference engine can be used to automate the smart decision at run time. Figure 2 shows the system flow of proposed inference engine. The proposed system will acquire the data from users, sensors, and vehicle control units and then forward it to the inference engine. The smart contract centric inference engine will analyze the input data, make decisions, and make plans according to the predefined rules. Then actuators will be used to act on the resolutions.

### 3.2. Data Flow with Inference Engine

**Figure 3:**
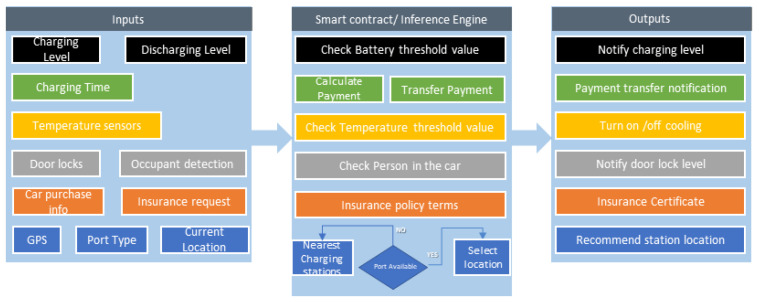
Data flow with inference engine.

Blockchain provides a record of tamper-resistant transactions. Every reading within the blockchain is timestamped and hard to tamper. Salimitari et al. [31] performed a survey of consensus protocols in the blockchain. They mentioned that a blockchain-based system is reliable and strong as its base consensus system, and hyper ledger fabric uses a practical Byzantine fault tolerance consensus method suitable for private blockchain. A smart contract is one of the main modules of blockchain. We have used smart contracts as an inference engine to make decisions. Heng et al. [32] used the smart contract for the run-time verification of sensing and actuating tasks. They also explain task management in the IoT environment. The sensor calls the smart contracts and sends recommendations to submit sensing functions and sensor ID+s and content. Smart contracts use device identifiers to verify that the sensor licenses are available on the blockchain. If the device is not registered in the network, access will not be allowed. The smart contractor analyzes the generated tracking data. The sensing details analyzed in the transaction proposal are used for comparison with the rules. Figure 3 shows the dataflow with smart contract centric inference engine. Once the conditions are met, the smart contract will issue an event informing the client that they can perform the following tasks; otherwise, they will warn the client that the conditions are not met. For example, a smart contract will take the charging and discharging level of the battery and check the threshold values predefined in the code. If the value is low from the threshold value, it will generate a notification to the car owner to refill the battery. Other tasks could be calculating the payment after charging, checking the car’s temperature, and finding the nearest charging station. The client assigns those tasks to the actuator.

### 3.3. Consensus in Private Blockchain

**Figure 4:**
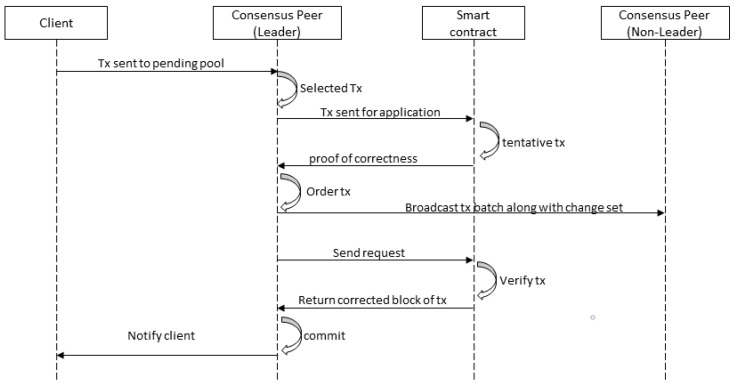
Sequence diagram for consensus in a private blockchain.

The consensus is one of the critical modules of blockchain; it plays a significant role in making blockchain a reliable platform [33]. Hyperledger Fabric provides a new way for consensus. The first step in the consensus process is to receive transactions from the client application. The transaction service requested to maintain the confidentiality of the transaction may not be recognized. In other words, the user can split or encrypt the contents of the transaction. Transactions are sent through the ordering peer. Depending on the consensus algorithm and configuration strategy, group transactions are processed to specify time limits or to limit the number of transactions allowed. Figure 4 shows the sequence diagram for consensus in a private blockchain.

For efficiency, the ordering peer does not perform a single transaction, but groups multiple transactions into one block. In this case, the ordering peer must accept and send a default array of transactions within each block, to confirm the transaction, the consensus is based on a smart contract layer. This is because transactions contain business logic. The smart contract layer verifies transactions by ensuring that each transaction complies with the strategy and contract specified in that transaction. Invalid transactions are rejected and can be deleted from any cluster within the cluster. Possible verification errors can be divided into two categories: syntax errors and logic errors. For syntax errors (e.g., invalid input, unverified signatures, and duplicate transactions), we need to drop the transaction. The second type of error is more complex, so it should focus on whether to continue processing, such as double-spend transactions or shipping errors. If necessary, for the strategy, these transactions can be recorded for review.

### 3.4. Transaction

**Figure 5:**
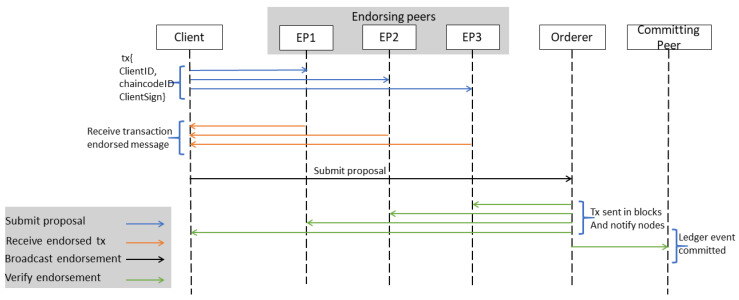
Sequence diagram for transaction flow.

The sequence diagram for transaction flow is explained in Figure 5. Client initiates a transaction(Tx) which contains ClientID, chaincodeID, txpayload, timestamp and client signature. Endorsing peers (EPs) verify the signature and execute the transaction. EPs check the redundancy and formation of transaction. They also check the validity of signatures through a membership service provider (MSP). After proper checking, EP adds their sign into Tx and sends it back as a proposal response. Proposal responses are inspected at the application level. After inspecting client assembles, this transaction proposal is broadcasted along with the response to the orderer peer. The ordering service is available upon request by orderers, and it can tolerate failure or lapse of nodes. In the end, the transaction is validated and committed by committer peers and update the ledger. The transactions per second (TPS) rate is higher in Hyperledger Fabric [34].

### 3.5. Machine Learning and Blockchain

The data received from the blockchain served as the input for the machine learning model. The machine learning model can be trained using Python. Blockchain data will be stored in CouchDB, which is the ledger state database used by each node. The trained model will then be used for real-time prediction and making decisions. The prediction results will be forwarded to the smart contract module of blockchain.

## 4. Architecture

**Figure 6:**
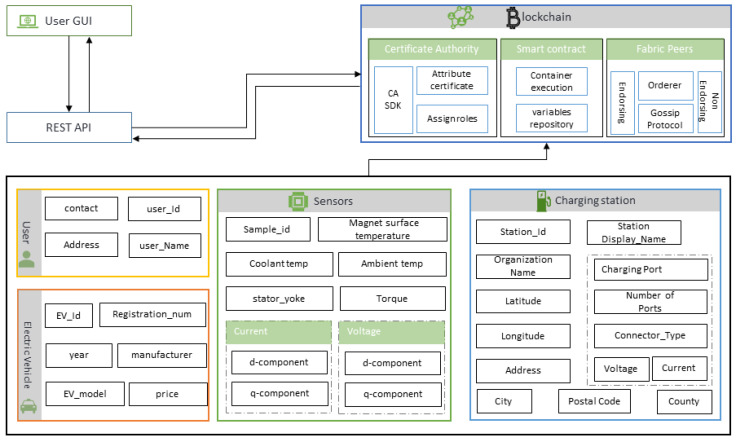
Blockchain based architecture.

In our proposed system, a smart contract takes the inputs from different sensors and users and makes decisions without human interference. Figure 6 shows the blockchain-based architecture of the proposed system. The user can be any of the participants, including the car owner, station manager, insurance agent, or regulator. The regulator will store the information related to EV, which might include unique id, registration number, model, and price. The vehicle control unit (VCU) is one of the core components of the EV system [35]. It takes the sensor’s information, including magnet surface temperature, coolant temperature, ambient temperature, torque, current, and voltage information. The regulator will also store the charging station information, including their latitude and longitude. A smart contract-based inference engine will take this information and notify the nearest charging station concerning EV’s location. Clients or users with a graphical user interface (GUI) can interact with blockchain using representational state transfer (REST) application program interface (API).

### 4.1. Vehicle Control Unit

**Figure 7:**
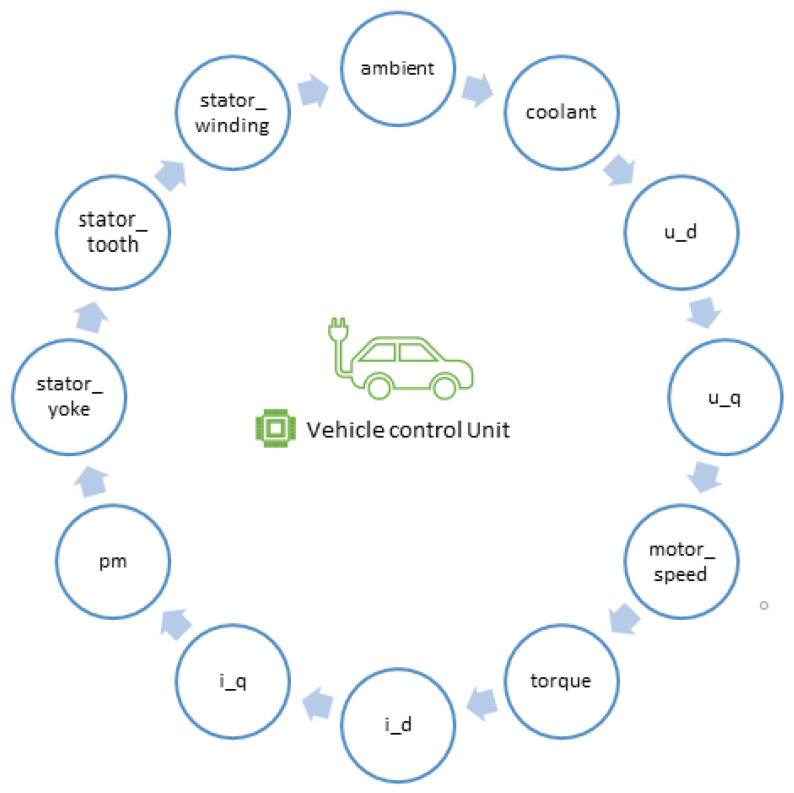
Components of vehicle control unit.

The vehicle control unit (VCU) is the core of the EV control system. VCU reads signals from sensors and then transmits this information to the database. It helps to gain intelligent and robust control of the electric vehicle. It is also called an electronic control unit. It is responsible for the recording of many sensors such as ambient temperature, coolant temperature, voltage ($u_{d},u_{q}$), motor speed, torque, current ($i_{d},i_{q}$), permanent magnet surface temperature (pm), stator yoke, and tooth temperature. Figure 7 depicts some of the sensor readings associated with VCU.

### 4.2. Blockchain

We have used an open-source platform Hyperledger Fabric by IBM for development purposes. Hyperledger Fabric is an enterprise-level, licensed distributed ledger technology platform. Compared with some other popular distributed ledger technologies or blockchain platforms, it provides some very critical differentiating capabilities.

In Fabric architecture, chaincode’s “read” operation to ledger obtains data through the state database. The default state database embedded in the Fabric is level dB, which provides simple key-value pairs operations. CouchDB offers excellent support for JSON format data and supports rich queries. CouchDB is used as the ledger state database by each peer. CouchDB makes queries more efficient by assigning indexes using chaincode, and it also allows the user to query more massive datasets. Some data are private and can only be stored on a specific peer, that will be deleted when certain conditions are met. Therefore, we have defined an individual data collection. According to the definition in the docker YAML file, each peer will connect to an independent CouchDB database [36]. The query command will get the endorsed result in the blockchain distributed ledger, but it will not generate or submit a transaction. The command invokes will create and endorse the transaction and submit it to the channel network. The consensus layer uses the communication layer to communicate with clients and other peers on the network [37]. According to the default consensus algorithm, all blocks must be checked through the peer nodes. The business network mainly consists of three actors, participants, assets, and transactions. Table 1 represents the business network components of the proposed blockchain-based system. Transactions are usually defined in the chaincode. Each peer has its independent state database, and its content “should” be the result of a consistent transaction endorsed. Chaincode does not directly modify the database for ledger’s “write” operations (add, update, delete). Instead, the client application first submits a proposal to several peers that meet the endorsement policy conditions. It is executed by chaincode, and the proposal response is obtained through the endorsing peer (endorsement node). After the client application verifies the same, the original request will be encapsulated as a transaction and submitted to the orderer. The orderer processes and distributes the transaction to all peers. Then the peer will update the state database. In this process, the transactions will be collected and packaged as a block and then added to the blockchain to become unchangeable content.

**Figure 8:**
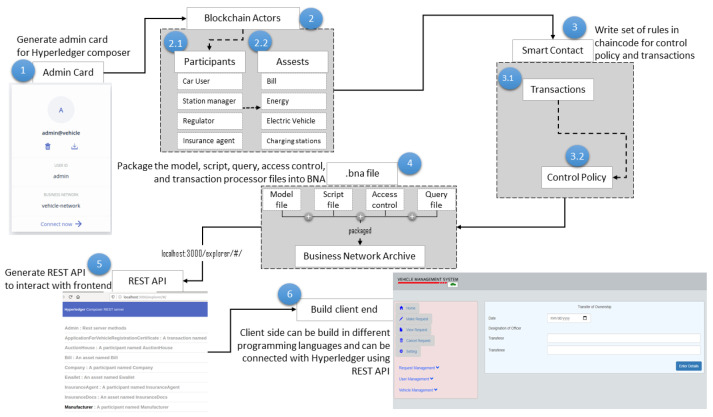
Process flow for private blockchain desgin.

In some scenarios, the blockchain needs to process some sensitive or private data. Only authorized specific peers can save, endorse, submit, and query them. Fabric provides a mechanism called private data and provides a series of specific APIs, such as chaincodestubinterface.putprivatedata, chaincodestubinterface.getprivatedata, are used. Through this mechanism, these data are only visible to authorized peers [38]. Unauthorized peers can not manipulate the hash value of this part of the data. Figure 8 explains the process flow for the designing of the private blockchain. Composer allows us to generate an admin card for each network. By using the admin card, we can define blockchain actors such as participants and assets. A set of rules is written in a smart contract. Then model, script, query, and access control files all are packages into the business network archive (.BNA). A REST API is generated using this BNA file to interact with the front end. The client-side can be built in different programming languages.

A large number of transactions are used in the designing of blockchain for EV management. Algorithm 1 shows the pseudo-code for one of those transactions. This migration transaction is defined in the smart contract. This function will be called automatically when two registered users want to interact with each other for selling of buying a car from each other without involving any third party. It will validate the user IDs of both buyer and seller from the certificate authority.
**Algorithm 1** Pseudo-code for migration transaction.**Ensure:** Initialize Smart contract**Ensure:** Seller and Buyer are valid registered user $SUser_{ID}$ = Seller ID $BUser_{ID}$ = Buyer ID **if**
EVState = ‘Available for sale’ **then**   **if**
UserState = buy **then**     $SEV_{ID}=SellOffer(EV_{ID})$     BuyOffer ($BUser_{ID},SEV_{ID})$     SellList -= $SEV_{ID}$     EVState = Sold   **end if**   EV has been transferred to User + ‘$BUser_{ID}$’ **else**   EV is not for Sale **end if**

The buyer will initiate a request stating that he wants to buy the specific car. If the EV state is available for sale, then the algorithm will proceed within the IF statement; otherwise, it will notify the user that it is not for sale. In the case of a successful transaction, the EV will be removed from the selling lists, and the EV state will be updated as sold so that the owner of this car will not receive the buying request from other users in the future.

### 4.3. Smart Contract

**Figure 9:**
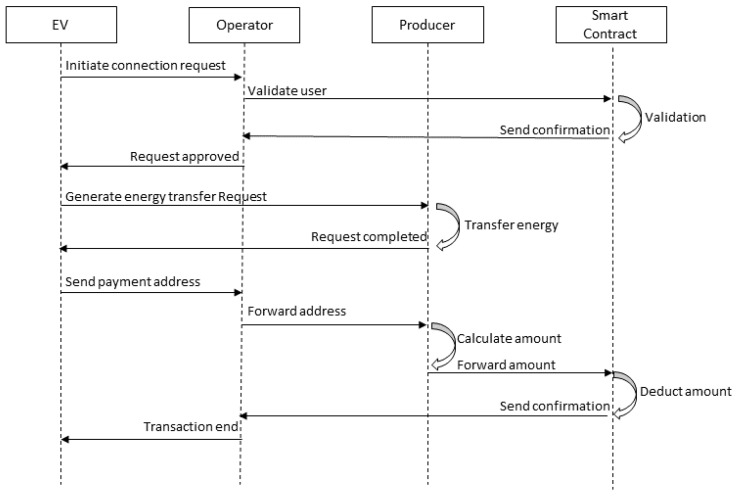
Sequence diagram for transaction using smart contract.

The smart contract often mentioned in blockchain technology is called chaincode in Fabric. It is a program that implements the chaincode interface (including init and invoke). It needs to be installed on the endorsing peer and is generally executed in a separate docker container. The sequence diagram for transaction flow using a smart contract is explained in Figure 9. After the client application sends a request, the chaincode init or invoke interface method will be called, return the execution result, and generate a read set and write set. Chaincode can read the state database, but cannot directly modify the state database, nor will it directly submit it to the orderer node. The result of the execution of the chaincode is not the final result. It is just a result of many nodes in this distributed system. Whether this result will be accepted by this blockchain network (channel) requires each endorsing peer to reach consensus. The chaincode interface provided by Fabric can support three development languages: go, node.js, and java.

**Figure 10:**
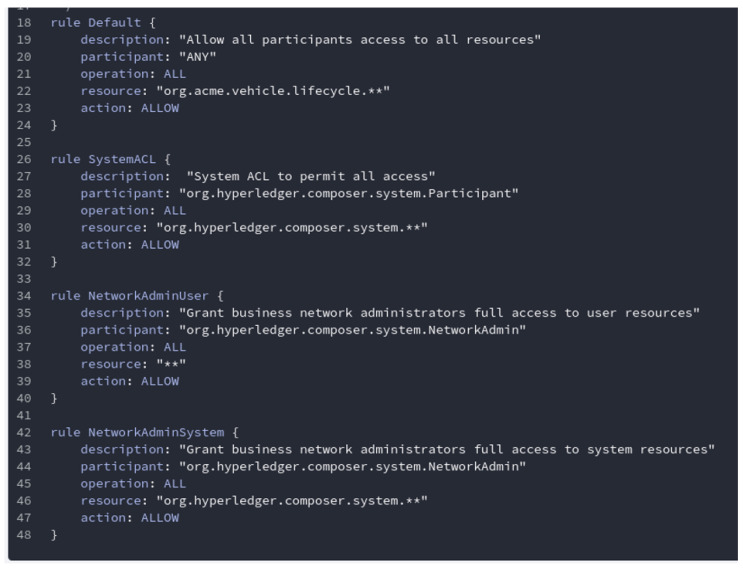
Control policy defined in smart contract.

Control policy is also defined in the smart contract. It allows user to regulate The privileges and maintain confidentiality and integrity of user-information. Access control policies gives specific access to only respective user. Figure 10 shows a snippet of control policies define in the smart contract.

### 4.4. Machine Learning

Machine learning (ML) is used to make the proposed smart contract-centric inference engine for intelligent electric vehicle transportation systems better and more reliable. Measuring the torque, rotor, and stator temperatures of the electromotor are not reliable nor economically feasible in commercial applications [39]. The temperature has a significant effect on the performance of the motor. The torque produced by the motor decreases in inverse proportion to the increased magnet temperature [40]. For the training purpose a permanent magnet synchronous motor (PMSM) open-source dataset is acquired [41], which contains 998,070 rows. We performed feature engineering and applied an optimized double-layer LSTM model [42]. We have used a genetic algorithm for optimal feature selection. We predicted the stator temperature by using the other available features in the dataset. The data are labeled and includes multiple predictor variables, so it is a multivariant, supervised ML problem. The response variable is type numeric, so we have used optimized bidirectional LSTM. Prediction of permanent magnet temperature is essential for the better and safe performance of PMSM [43]. LSTM has cells that are effective in predicting the sequences and handle the long-term temporal dependencies. It has a set of gates, input, output, and forget gate [44]. Recurrent neural networks with short-term memory can learn longer observation sequences. LSTM can be modeled almost entirely with various input variables, and it is the main advantage for the prediction of time series. This is because traditional linear methods can be challenging to adapt to multivariate prediction problems or multiple inputs. LSTM overcomes vanishing gradient problems and eventually disappears by reverse replication. Therefore, it exactly matches the time series forecasting problems.

The logistic sigmoid function σ serves typically as the activation function of individual gates, and *t* is used for the current interval. Equation (1) determines the function for the input gate. Among them, $W_{i}$ depicts the weight for input, and $b_{i}$ describes the bias vector. Equations (2) and (3) show functions for output gate and forget gate, respectively. $h_{t-1}$ serves the hidden state as current time in these Equations [8].

(1) $$I_{g}=\sigma \left(W_{i}\times \left(h_{t-1},x_{t}\right)+b_{i}\right)$$

(2) $$O_{g}=\sigma \left(W_{o}\left(h_{t-1},x_{t}\right)+b_{o}\right)$$

(3) $$F_{g}=\sigma \left(W_{f}\times \left[h_{t-1},x_{t}\right]+b_{f}\right)$$

**Figure 11:**
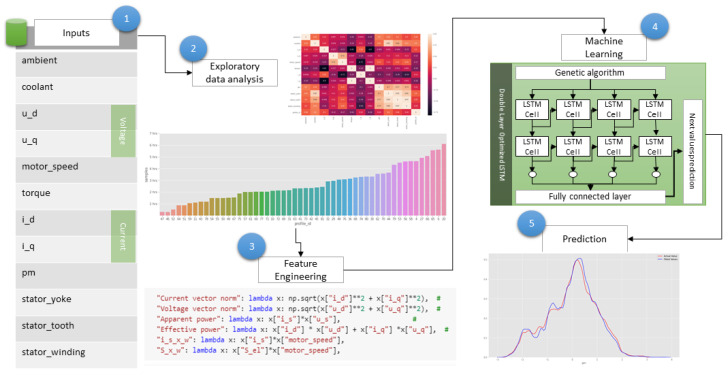
Process flow chart for prediction using machine learning.

Figure 11 shows the process flow chart for prediction using machine learning. It starts by taking the input variables and doing exploratory data analysis. Table 2 shows the input variables, their explications and description. After data analysis feature engineering is performed, we added new variable for current vector normalization (Equation (4)), voltage vector normalization (Equation (5)), apparent power (Equation (6)), and effective power (Equation (7)). Where λ(x) is used to define for domain or range. Then we applied the genetic algorithm to get optimal features. We pass those optimal parameters to the double layer LSTM, and we got the output in the form of a prediction.

(4) $$i_{s}=\lambda \left(x\right):\sqrt{x{\left(i_{d}\right)}^{2}+x{\left(i_{q}\right)}^{2}}$$

(5) $$u_{s}=\lambda \left(x\right):\sqrt{x{\left(u_{d}\right)}^{2}+x{\left(u_{q}\right)}^{2}}$$

(6) $$P_{a}=\lambda \left(x\right):\left(x\left(i_{s}\right)\times x\left(u_{s}\right)\right)$$

(7) $$P_{e}=\lambda \left(x\right):\left(x\left(i_{d}\right)\times x\left(u_{d}\right)+x\left(i_{q}\right)\times x\left(u_{q}\right)\right)$$

Since all the variables’ features are measured over time and are time-dependent, the later analysis is done assuming the data to be time-series data. Each row represents one snapshot of sensor data at a certain time step. The sample rate is 2 Hz (one row per 0.5 s). distinctive sessions are identified with profile ID. There are 52 unique profiles.

## 5. Results

This section consists of the evaluation results of the proposed system. The simulation environment used during the experimental phase is summarized in Table 3. Version 1.4.1 of Hyperledger Fabric is used to define the business network. We performed the simulations on Ubuntu Linux 18.04.1 LTS operating system.

**Figure 12:**
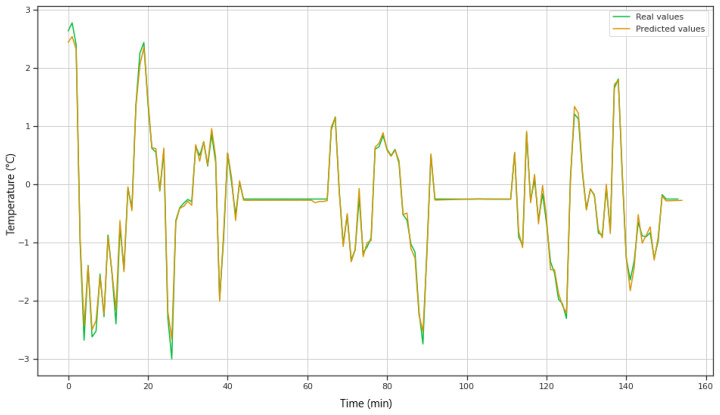
Prediction graph with actual and forecasted values.

The machine learning part was used to get the stator temperature of eclectic motor. The predicting value was used by inference engine. Figure 12 shows the comparison of actual and predicted stator temperature.

We also evaluated the blockchain network to analyze the performance of our proposed system. To assess the effectiveness of the proposed EV-blockchain system, various experiments were performed using multiple performance methods. To do this, we use IBM’s open-source simulator called Hyperledger Caliper [45]. For testing, we created three groups of 250, 500, 750 sections. We recorded at least a percentage of the delay (in milliseconds) at the maximum and maximum time on the proposed blockchain platform.

**Figure 13:**
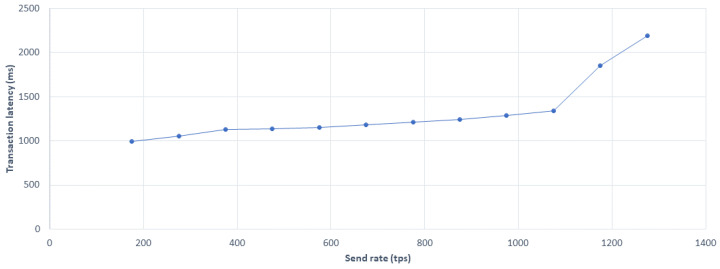
Average transaction latency evaluation.

Figure 13 shows the average transaction delay with an increase in transaction latency. Equation (8) was used to calculate latency $L_{t}$, where $T_{c}$ is the confirmation time, $N_{t}$ is network threshold, and $T_{s}$ represents submission time. This happened at the increment in the request of the user on the network latency also increased. After the transmission rate of optimal tps, also called as best transmission rate, the delay began to increase drastically.

(8) $$L_{t}=T_{c}\times N_{t}-T_{s}$$

**Figure 14:**
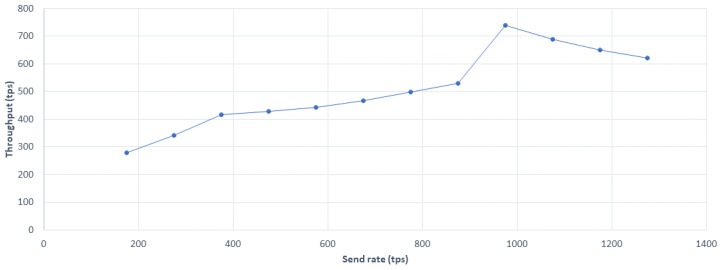
Average transaction throughput evaluation.

Figure 14 depicts the average transaction throughput evaluation. Equation (9) was used to calculate transaction throughput $TP_{t}$, where $V_{t}$ is the total valid transactions and *T* represents total time in seconds.

(9) $$TP_{t}=\frac{\sum (V_{t})}{\sum (T)}$$

**Figure 15:**
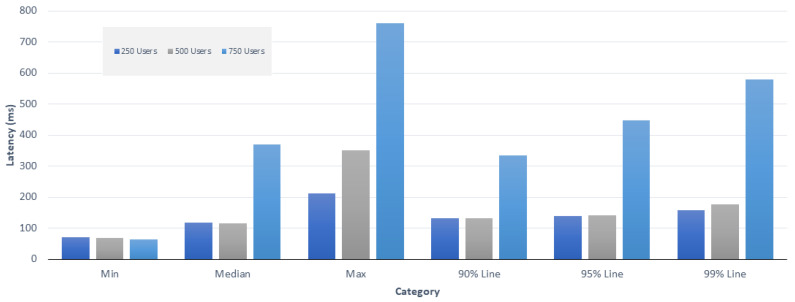
Get request transaction latency evaluation.

The graph in Figure 15 shows a bar graph to visualize the network delay depending on the number of users. The median values observed were 117, 115, and 369 ms for the group of 250, 500, 750 users, respectively. The highest values were 213, 352, and 761 ms, respectively. Get request transaction latency studies showed that delays in the system increased as the number of users increased. The number of concurrent transactions that Hyperledger Fabric could handle depended on the number of nodes in the network. If there were six nodes in the network, it could handle up to 10,000 concurrent transactions [46].

The use of resources was analyzed to determine CPU and memory usage. It also records 12 incoming and outgoing traffic through the proposed system. Table 4 explains the proposed method’s resource utilization analysis. It shows the max and average memory and CPU consumption. It also displays incoming and outgoing traffic. Resource distribution did not apply to vehiclenetwork_peer_1 as it did not use more memory or network-related resources. The administration was usually running a network, so it took more resources and was used as the coach database system database. Traffic, CPU usage, and memory resources were not kept high or low, so it could easily control many users. The results of the resource analysis showed that the share of the proposed private blockchain was entirely high. Low memory usage and low traffic provided a comfortable and stable user experience.

## 6. Conclusions

In this paper, we have illustrated the novel intelligent transportation system for electric vehicles using state of the art technologies such as blockchain and machine learning. The EV management system is improved by using the blockchain features such as transparency by tracking the previous transactions and tamper-resistant property to improve the system reliability. Our proposed solution helps to overcome the hassle of paying for electric vehicles charging and managing trust and privacy in various electric vehicle databases. It creates a decentralized environment in which all the actors can trust each other. The first module is developed using a private blockchain to ensure the transparency and stability of the registry. Blockchain is transparent as it can track the provenance of the previous transactions, and the tamper-resistant property of blockchain makes it reliable. The second module is developed using an improved dual-layer LSTM algorithm. We have used the optimized machine learning algorithm to predict the stator temperature. Prediction results, transaction latency, and throughput evaluation demonstrate the proposed system’s robustness and reliability. In the future, we will add cryptocurrency to add prepaid and postpaid mechanisms to pay for EV charges and we will also work on improving the response time.

## Figures and Tables

**Table 1:** Blockchain business network components.

Assets	Participants	Transactions
Bill	Car User	CreateWallet()
Energy	Station manager	QueryWallet()
Electric Vehicle	Regulator	TopupWallet()
Charging stations	Insurance agent	LogTransaction()
	Admin	UpdateTransaction()
		Migration()
		FuelPyamentRecord()
		EvProfileRegistered()
		getMyEnergyRequirements()
		storeSchedule()

**Table 2:** Input variables along with description.

SR #	Variable	Explication	Description
1	ambient	Ambient temperature	Ambient temperature as measured by a thermal sensor located closely to the stator
2	coolant	Coolant temperature.	Coolant temperature. The motor is water cooled. Measurement is taken at outflow.
3	u_d	d-component	Voltage
4	u_q	q-component	Voltage
5	motor_speed	Motor speed	PMSM motor speed
6	torque	Torque	Torque induced by current.
7	i_d	d-component	Current
8	i_q	q-component	Current
9	pm	Permanent magnet surface temperature	PM representing the rotor temperature. This was measured with an infrared thermography unit.
10	stator_yoke	Stator yoke temperature	Stator yoke temperature measured with a thermal sensor.
11	stator_tooth	Stator tooth temperature	Stator tooth temperature measured with a thermal sensor
12	tator_winding	Stator winding temperature	Stator winding temperature measured with a thermal sensor
13	profile_id	Unique profile	Each measurement session has a unique ID.

**Table 3:** EV-blockchain simulation environment.

Sr #	Component	Description
1	Processing Unit	Intel Core i5-8500 @ 3.00GHz
2	Random Access Memory	16 GB
3	OS	Ubuntu Linux 18.04.1
4	Docker Composer	v 1.24.0
5	Docker Engine	v 18.06.1
6	Node	v 8.11.4
7	Python	v 3.6
8	Hyperledger Fabric	v 1.4×
9	Development environment	Fabric-playground
10	Command line tool	Fabric composer-cli

**Table 4:** Resource utilization analysis of proposed system.

Type	Name	Memory (max)	Memory (avg)	CPU (max)	CPU (avg)	Traffic In	Traffic Out
Process	Node local-client.js	101.9 MB	96 MB	24.31%	13.1%	-	-
Docker	dev-peer0.regulator.com	9.3 MB	7.7 MB	2.1%	1.01%	541.3 KB	292.6 KB
Docker	dev-peer1.regulator.com	8.6 MB	8.4 MB	6.73%	3.96%	3.0 MB	1.6 MB
Docker	dev-peer0.shop	5.8 MB	6.2 MB	0%	0%	506 B	0 B
Docker	dev-peer1.shop	7.3 MB	8.6 MB	6.46%	3.92%	1.9 MB	913.2 KB
Docker	peer1.org1.pump.com	75.2 MB	97.5 MB	21.64%	15.29%	4.98 MB	9.7 MB
Docker	peer0.org1.pump.com	106.1 MB	114 MB	12.91%	9.88%	5.13 MB	801.3 KB
Docker	vehiclenetwork_peer_1	0 B	0 B	0.00%	0.00%	0 B	-
Docker	ca_peerOrg2	18.5 MB	16.1 MB	0.00%	0.00%	0 B	-
Docker	orderer.vehicle.com	42.1 MB	41.2 MB	9.06%	6.25%	5.1 MB	14.6 MB
Docker	vehiclenetwrok_ca_1	8.6 MB	9.3 MB	0.00%	0.00%	0 B	0 B
Docker	ca_peerOrg2	4.4 MB	5.4 MB	0.00%	0.00%	0 B	0 B
Docker	couchdb.org1.pump.com	98 MB	89 MB	8.49%	4.70%	180.4 KB	296 KB
Docker	couchdb.org2.pump.com	92 MB	85 MB	7.00%	5.31%	302.6 KB	152 KB

## Abbreviations

The following abbreviations are used in this manuscript:

EV	Electric vehicles
LSTM	Long short term memory
DC	Direct current
PMSM	Permanent magnet synchronous motor
ITS	Intelligent transportation system
IEVTS	Intelligent electric vehicle transportation system
VCU	Vehicle control unit
SDN	Software-defined network
PoW	Proof of work
QoS	Quality of service
BNA	Business network archive
GUI	Graphical user interface
REST	Representational state transfer
API	Application program interface
EP	Endorsing peers
MSP	Membership service provider
TPS	Transactions per second
ML	Machine learning
CPU	Central processing unit
